# Testing and Analysis Method of Low Remanence Materials for Magnetic Shielding Device

**DOI:** 10.3390/ma16020681

**Published:** 2023-01-10

**Authors:** Yuan Cheng, Yaozhi Luo, Ruihong Shen, Deyu Kong, Weiyong Zhou

**Affiliations:** 1 College of Civil Engineering and Architecture, Zhejiang University, Hangzhou 310027, China; 2 China United Engineering Co., Ltd., Hangzhou 310022, China; 3 College of Civil Engineering, Zhejiang University of Technology, Hangzhou 310023, China; 4 School of Instrumentation Science and Optoelectronics Engineering, Beihang University, Beijing 100191, China

**Keywords:** magnetic shielding device, low remanence material, extremely weak magnetic field

## Abstract

Magnetic shielding devices with a grid structure of multiple layers of highly magnetically permeable materials (such as permalloy) can achieve remanent magnetic fields at the nanotesla (nT) level or even lower. The remanence of the material inside the magnetic shield, such as the building materials used in the support structure, can cause serious damage to the internal remanence of the magnetic shield. Therefore, it is of great significance to detect the remanence of the materials used inside the magnetic shielding device. The existing test methods do not limit the test environment, the test process is vulnerable to additional magnetic field interference and did not consider the real results of the material in the weak magnetic environment. In this paper, a novel method of measuring the remanence of materials in a magnetic shielding cylinder is proposed, which prevents the interference of the earth’s magnetic field and reduces the measurement error. This method is used to test concrete components, composite materials and metal materials commonly applicated in magnetic shielding devices and determine the materials that can be used for magnetic shielding devices with 1 nT, 10 nT and 100 nT as residual magnetic field targets.

## 1. Introduction

Large-scale magnetic shielding devices, used high permeable magnetic materials and high conductivity materials, such as permalloy and aluminum, to prevent the external magnetic field from entering its interior, based on the flux shunt effect [1,2,3] and eddy current effect [4,5], are used to provide extremely weak magnetic field environment. In the weak magnetic environment, many scholars have carried out frontier work, such as the measurement of biological signals such as magnetocardiography and magnetoencephalography [6,7,8], the measurement of geophysical research samples [9], the measurement of electric dipole moments [10], research on high-precision magnetic measuring instruments, such as serf atomic gyroscopes and magnetometers [11,12] and so on. Since the internal magnetic field of the magnetic shielding device is very small, the remanence of internal supporting structures (such as platforms for placing instruments) and scientific instruments equipment (such as cardio brain magnetic equipment) may have a greater impact on the internal magnetic field, so the remanence of the magnetic shielding device should be analyzed when selecting the supporting material of the magnetic shielding device.

Borradaile et al. [13] put the tested sample into a square container and used Molspin “BigSpin” magnetometer to test the remanence of the material. Feinberg et al. [14] used DC-SQUID sensors to design a three-axis remanence to test the low-temperature remanence and magnetism of magnetic minerals and provide explanations for geophysical phenomena such as core dynamics and paleoclimate. Bilardello et al. [15] tested the hysteresis loop of rock samples in geology through vibrating sample magnetometer (VSM), and analyzed the remanence and other magnetic properties. He et al. [16] improved the low-temperature calibration system of the Hall probe and established a low-temperature remanence measurement system for bulk permanent magnets, which can accurately test the remanence performance of bulk permanent magnets. Biedermann et al. [17] used a vibrating sample magnetometer to test the hysteresis and thermomagic of rock samples. It is used to determine the saturation magnetization, coercivity and remanence of rock samples to analyze the influence of seismic slip-on rock magnetism. Robustelli et al. [18] measured the Thermomagnetic susceptibility curves of the sample by heating the sample to 700 °C, and then cooling it to room temperature to measure the magnetic signature along interpolate sheet zones. Lund et al. [19] tested the hysteresis loop of deep-sea sediment samples through the MicroMag cycle. The remanence, saturation magnetization and coercivity were calculated to estimate the particle size distribution and percentage of ferromagnetic minerals and titanomagnetite minerals. Monaico et al. [20] studied the magnetization curve of coaxial core–shell magnetic nanostructures with vibrating sample magnetometer, analyzed the influence of different Ni content on remanence, squareness ratio, and coefficient. Wei et al. [21] tested the remanence in the iron core of the power transformer through the LCR tester and predicted the remanence through the relationship between it and the measured magnetization inductance.

For previous studies, the main purpose is to infer geological changes based on the magnetic analysis of rocks, there are mainly two methods to test the remanence of materials: one is to obtain the material’s remanence properties by testing the material’s hysteresis loop, which tests the magnitude of remanence in relation to the magnetization magnitude and cannot accurately reflect the material’s remanence during application. Additionally, the other is to test the magnetic field near the material directly by a magnetometer to obtain the material’s remanence value, but the existing methods for measuring the remanence of materials do not limit the testing environment, and they are directly tested in the earth’s magnetic field. Because the earth’s magnetic field has hundreds of nT fluctuations, when measuring the remanence of materials less than hundreds of nT, it is impossible to accurately calculate the remanence of materials according to the change of the fluxgate value, which leads to large test errors. Furthermore, the previous test results of material remanence did not consider the real results of the material in the weak magnetic environment, but the purpose of this study is to analyze the remanence of building and support structure materials used in the magnetic shielding device, so it is not applicable. In order to select the building materials and support materials that can be used in large-scale extremely weak magnetic field magnetic shielding devices, this paper uses a fluxgate magnetometer to test the material samples. The test process is carried out in a magnetic shielding cylinder to eliminate the interference of the external magnetic field. Firstly, test the internal environmental magnetic field of the magnetic shielding cylinder, and then test the magnetic field around the material. The true remanence of the material is the magnetic field around the material minus the internal environmental magnetic field. The method proposed in this paper avoids the interference of the earth’s magnetic field and other magnetic fields, improves the measurement accuracy, and can directly test the remanence of materials in a weak magnetic environment, it is significant to analyze the materials of building and support structure inside the magnetic shielding device.

The rest of this paper is arranged as follows. In Section 2, the principles of testing and calculation of material remanence are first introduced, followed by the testing procedures. Section 3 presents the test results and discussion. Section 4 summarizes brief conclusions.

## 2. Principle of Remanence Measurement

In this paper, a fluxgate magnetometer (CTW-6M, resolution: 0.2 nT, range: 100,000 nT) is used to test the remanence of materials commonly used in magnetic shielding devices (concrete materials, composite materials and metal materials), during the test, the material is placed in a magnetic shielding cylinder to cancel the effect of the Earth’s magnetic field influences. 

The remanence of a material $B_{r}$ is the absolute value of the test magnetic field $B_{T}$ minus the background magnetic field $B_{B}$:

(1) $$B_{r}=\left|B_{T}-B_{B}\right|$$

where $B_{r}$ is the material remanence, $B_{T}$ is the test magnetic field, $B_{B}$ is the background magnetic field (including the environmental magnetic field in the barrel and the sensor bias).

The magnetic shielding cylinder used in the test has a four-layer permalloy structure. The remanence of the magnetic shielding cylinder can be calculated by the magnetic shielding factor *S*:

(2) $$B_{B}=\frac{B_{0}}{S}$$

where $B_{0}$ is the Earth’s magnetic field.

For the shielding coefficient of a single-layer shielding cylinder, the formula can be calculated as follows [22]:

(3) $$S=\frac{2\mu tR^{\frac{1}{2}}}{L^{\frac{3}{2}}}$$

where S is the shielding coefficient; μ is the relative permeability; R is the radius of the shielding layer; t is the thickness of the shielding layer; L is the length of the shielding layer.

The formula for calculating the shielding factor of the multi-layer magnetic shielding cylinder is:

(4) $$S_{z}=S_{n}\prod_{i=1}^{n-1}S_{i}\left[1-{\left(\frac{D_{i+1}}{D_{i}}\right)}^{j}\right]$$

where $S_{z}$ is the *n*-layer shielding coefficient; $S_{i}$ is the *i*-th layer shielding coefficient (the outermost is the first layer).

**Figure 1:**
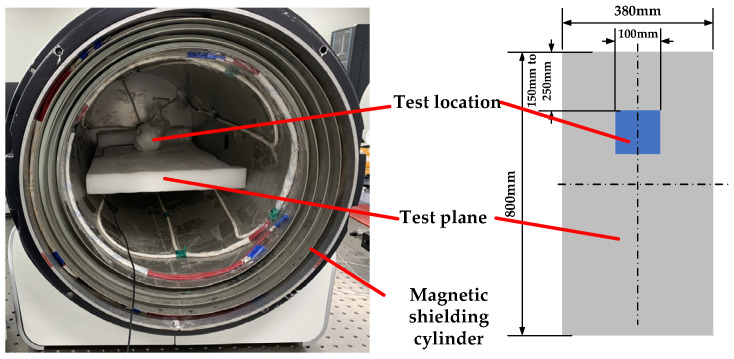
Schematic diagram of the test position in the magnetic shielding cylinder.

Since the magnetic shielding cylinder is a long strip structure, there is a gradient in the internal magnetic field. The area with small magnetic field in the magnetic shielding cylinder is determined as the test area through the experimental test. It can be seen from the results that the closer to the shielding layer, the lower the internal remanence, but due to the magnetic concentration of permalloy, it cannot be infinitely close to the cylindrical wall. Therefore, choosing a location 150 mm to 250 mm from the bottom of the magnetic shielding cylinder for testing, as shown in Figure 1.

**Figure 2:**
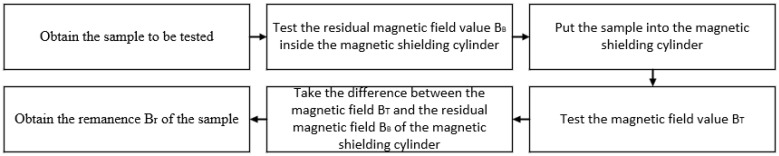
Schematic diagram of test process.

When the test area in the magnetic shielding cylinder is determined, the test flow diagram is shown in Figure 2, and the test steps are as follows:

(1) Turn on the fluxgate magnetometer and preheat for 10 min;

(2) Open the door of the magnetic shielding cylinder, place the magnetometer probe on the test platform inside the magnetic shielding cylinder, and move the probe to the center;

(3) Close the door of the magnetic shielding cylinder and test the internal magnetic field BB of the magnetic shielding cylinder;

(4) Open the door of the magnetic shielding cylinder and place the sample to be tested on the head of the magnetometer probe;

(5) Close the door of the magnetic shielding cylinder and test the current situation of the magnetic field Bt inside the magnetic shielding cylinder;

(6) Calculate the material remanence by Formula (1);

(7) Repeat (1)–(6) to test the next sample.

**Figure 3:**
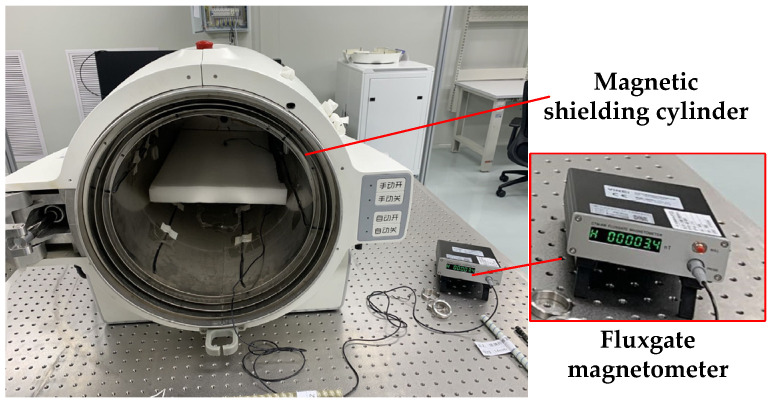
Magnetic shielding cylinder and fluxgate magnetometer.

The test device is shown in Figure 3.

## 3. Test Results and Discussion

### 3.1. Remanence Test of Concrete Component Materials

**Figure 4:**
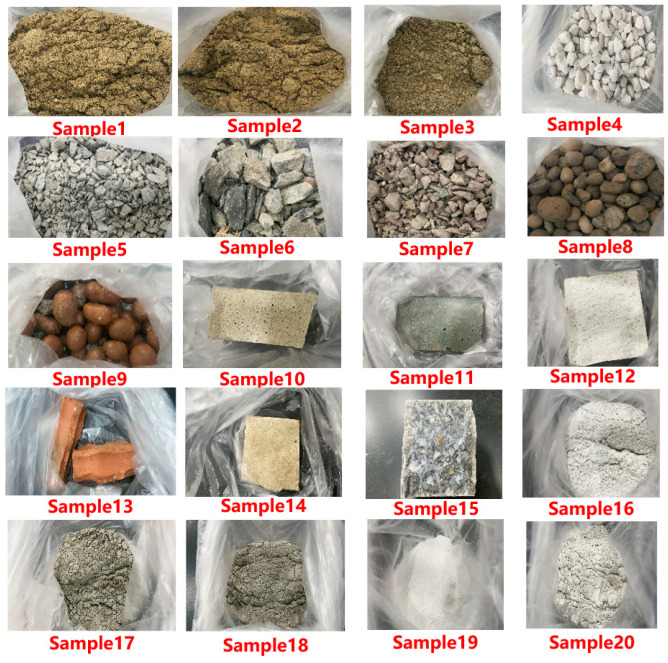
Test samples, sample 1 (sand from Hangzhou), sample 2 (sand from Jiande), sample 3 (sand from Ganjiang),sample 4 (dolomite), sample 5 (tuff), sample 6 (limestone), sample 7 (cement gravel), sample 8 (clay ceramsite from Jiaxing), sample 9 (clay ceramsite from Hubei), sample 10 (mortar block), sample 11 (grey cement mortar block), sample 12 (white cement mortar block), sample 13 (red brick), sample 14 (ceramic sheet), sample 15 (concrete block), sample 16 (white cement), sample 17 (grey cement), sample 18 (fly ash), sample 19 (quartz sand), sample 20 (mineral powder).

Concrete is often used as the supporting structure of large magnetic shielding devices. According to building requirements, the remanence test is carried out on some commonly used stones and gravels in concrete. The test samples are shown in Figure 4:

Following the test steps described above, the results are show in Table 1:

### 3.2. Remanence Testing of Composite Materials

**Figure 5:**
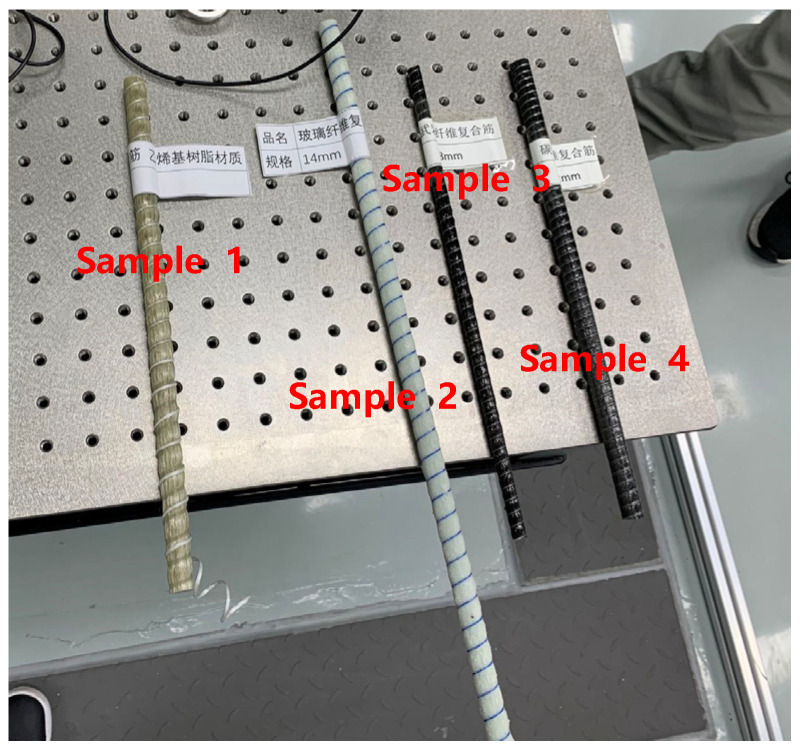
Test samples, sample 1 (vinyl resin), sample 2 (glass fiber), sample 3 (basalt fiber composite bar), sample 4 (carbon fiber composite bar).

Composite materials are commonly used as reinforcement for large magnetic shielding devices. According to the building requirements, the remanence test is carried out on the composite bars. The test sample is shown in Figure 5:

Following the test steps described above, the results are show in Table 2:

### 3.3. Remanence Test of Metal Structural Materials

**Figure 6:**
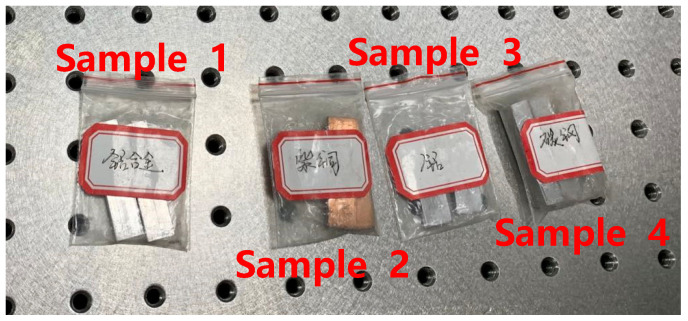
Test samples, sample 1 (aluminum alloy), sample 2 (copper), sample 3 (pure aluminum), sample 4 (carbon steel).

Metal materials are commonly used as internal supporting structures and supporting grids of large magnetic shielding devices. According to the building requirements, the remanence test is carried out on the metal structural materials. The test sample is shown in Figure 6:

Following the test steps described above, the results are show in Table 3:

### 3.4. Discussion

Different research objects have different requirements for the internal weak magnetic environment of the magnetic shielding device. For magnetocardiography and magnetoencephalography, the internal magnetic field of the magnetic shielding device is often required to be less than 1 nT. For instruments such as serf gyroscopes, the internal magnetic field of the magnetic shielding device needs to be less than 10 nT. For animal and plant weak magnetic environment experiments, the internal magnetic field of the magnetic shielding device needs to be less than 100 nT. Therefore, the magnetic shielding devices of different objects need to be constructed with appropriate remanence and corresponding materials (such as external support materials, internal structural materials).

According to the above requirements for the use of the internal space of the magnetic shielding device, when selecting an application area, it is common to select a vertical distance (usually 0.2 m to 0.5 m) from the materials described in this paper. According to the test results, if the magnetic field values of 1 nT, 10 nT and 100 nT is to be reached in the magnetic shielding device, the remanence of the supporting materials shall be lower than 5 nT, 50 nT and 140 nT, respectively, in the distance between the above materials.

Therefore, the materials that can be used for ≤1 nT space after comparison are show in Table 4:

The materials that can be used in the space of ≤10 nT are show in Table 5 (Materials other than those listed in Table 4):

The materials that can be used in the space of ≤50 nT are show in Table 6 (Materials other than those listed in Table 4 and Table 5):

In summary, the concrete composites with low remanence are concrete composites sample 2 (sand from Jiande), sample 6 (limestone), sample 12 (white cement mortar block), sample 16 (white cement) and sample 20 (mineral powder), the composite material with lower remanence is composite material sample 1 (vinyl resin), and the metal structure material with lower remanence is metal structure material sample 3 (pure aluminum). After obtaining the remanence value of the material, the corresponding magnetic field strength can be calculated through the vacuum permeability $\mu_{0}$ and substituted into the finite element simulation calculation, which can evaluate the influence of the remanence of the internal materials on the shielding device and improve the accuracy of the simulation calculation of the magnetic shielding device.

## 4. Conclusions

In order to reduce the adverse effects to the weak magnetic environment caused by the materials of the support and platform (including concrete member materials (stone, sand), composite materials and metal materials) in the extremely weak magnetic field, this paper proposes a method to test the remanence of materials by placing the tested samples in the magnetic shielding cylinder and using a fluxgate magnetometer. This method first tests the internal magnetic field of the magnetic shielding cylinder, and then the magnetic field around the material. The true remanence of a material is the magnetic field around the material minus the internal environmental magnetic field. This method can eliminate the interference of the external magnetic field on the test and obtain more accurate results. Through the test and analysis in this paper, in the 1 nT environment, concrete materials such as dolomite, white cement mortar block and white cement, composite materials such as vinyl resin and glass fiber, and metal materials such as aluminum alloy and copper can be used. In the 10 nT environment, concrete materials such as limestone, clay ceramsite from Jiaxing, and ceramic sheet can be used, and in the 100 nT environment, concrete materials such as cement gravel, grey cement mortar block, and fly ash can be used. Furthermore, the simulation accuracy of magnetic shielding device can be improved by combining test results of material remanence with finite element simulation and provide a basis for the structural support materials and platform materials used for the magnetic shielding device.

## Figures and Tables

**Table 1:** Concrete material remanence test results.

Test Sample	Test Magnetic Field B_T_ (nT)	Background Magnetic Field B_B_ (nT)	Remanence Br (nT)
Sample 1	−27.7	6.4	34.1
Sample 2	24.2	6.4	17.8
Sample 3	−91.6	6.4	98.0
Sample 4	41.8	6.4	0.4
Sample 5	−168.2	6.4	174.6
Sample 6	−1.8	6.4	8.2
Sample 7	−39.2	6.4	45.6
Sample 8	12.7	6.4	6.3
Sample 9	−13.4	6.4	19.8
Sample 10	80.3	6.4	73.9
Sample 11	35.4	6.4	29.0
Sample 12	3.3	6.4	3.1
Sample 13	−1244.2	6.4	1250.6
Sample 14	−1.0	6.4	7.4
Sample 15	−1005.2	6.4	1011.6
Sample 16	5.6	5.3	0.3
Sample 17	19.9	5.3	14.6
Sample 18	−31.2	5.3	36.5
Sample 19	6.5	5.3	1.2
Sample 20	4.3	5.3	1.0

**Table 2:** Composite materials remanence test results.

Test Sample	Test Magnetic Field B_T_ (nT)	Background Magnetic Field B_B_ (nT)	Remanence Br (nT)
Sample 1	3.5	3.4	0.1
Sample 2	3.8	3.4	0.4
Sample 3	3.8	3.4	0.4
Sample 4	4.1	3.4	0.7

**Table 3:** Metal structural materials remanence test results.

Test Sample	Test Magnetic Field B_T_ (nT)	Background Magnetic Field B_B_ (nT)	Remanence Br (nT)
Sample 1	3.8	3.4	0.4
Sample 2	3.9	3.4	0.5
Sample 3	3.7	3.4	0.3
Sample 4	8013.1	3.4	8009.7

**Table 4:** Materials available inside the space of ≤1 nT.

Test Sample	Remanence Br (nT)
Concrete component material sample 4 (dolomite)	0.4
Concrete component material sample 12 (white cement mortar block)	3.1
Concrete component material sample 16 (white cement)	0.3
Concrete component material sample 19 (quartz sand)	1.2
Concrete component material sample 20 (mineral powder)	1.0
Composite materials sample 1 (vinyl resin)	0
Composite materials sample 2 (glass fiber)	0.4
Composite materials sample 3 (basalt fiber composite bar)	0.4
Composite materials sample 4 (carbon steel)	0.7
Metal structural materials sample 1 (aluminum alloy)	0.4
Metal structural materials sample 2 (copper)	0.5
Metal structural materials sample 3 (pure aluminum)	0.3

**Table 5:** Materials available inside the space of ≤10 nT.

Test Sample	Remanence Br (nT)
Concrete component material sample 6 (limestone)	8.2
Concrete component material sample 8 (clay ceramsite from Jiaxing)	6.3
Concrete component material sample 14 (ceramic sheet)	7.4
Concrete component material sample 17 (grey cement)	14.6

**Table 6:** Materials available inside the space of ≤100 nT.

Test Sample	Remanence Br (nT)
Concrete component material sample 1 (sand from Hangzhou)	34.1
Concrete component material sample 2 (sand from Jiande)	17.8
Concrete component material sample 7 (cement gravel)	45.6
Concrete component material sample 9 (clay ceramsite from Hubei)	19.8
Concrete component material sample 11 (grey cement mortar block)	29.0
Concrete component material sample 18 (fly ash)	36.5

## Data Availability

Not applicable.
